# A Preliminary Study on the Improvement of Gangue/Tailing Cemented Fill by Bentonite: Flow Properties, Mechanical Properties and Permeability

**DOI:** 10.3390/ma16206802

**Published:** 2023-10-22

**Authors:** Hongsheng Wang, Dengfeng Chen, Ruihong Guo, Jiahao Tian, Bin Li

**Affiliations:** 1 School of Energy Engineering, Xi’an University of Science and Technology, Xi’an 710054, China; cumtwhs@xust.edu.cn (H.W.); 18191579210@163.com (R.G.); 22203077031@stu.xust.edu.cn (J.T.); lbblibin@163.com (B.L.); 2 Institute of Rock Burst Prevention and Control, Xi’an University of Science and Technology, Xi’an 710054, China

**Keywords:** bentonite, cemented backfill body, flow performance, bleeding rate, mechanical properties, permeability

## Abstract

Backfill mining has significant advantages in safe mining, solid waste utilization and ecological environmental protection, but solid waste materials (tailings, gangue and coal gasification slag, etc.), as derivative residues of the chemical and metallurgical industries, contain a large number of heavy metal elements, which is posing great challenges to the underground environment after backfill. In order to study the feasibility of bentonite for reducing the permeability of gangue/tailing sand cemented backfill body, relevant tests were carried out from the basic performance index, flow performance and mechanical properties of paste backfill materials. The test results show that bentonite has a significant effect on the water secretion rate of cemented fillers, and also promotes the improvement of slump and diffusion diameter of backfill slurry. The enhancement effect of mechanical properties in the early stage is not obvious, mainly concentrated in the middle and late stages of specimen curing. With the increase of bentonite content, the 28-day uniaxial compressive strength increased from 7.1 MPa and 7.9 MPa to 8.7 MPa and 9.0 MPa, respectively. Bentonite is filled between the pores of the cemented backfill with its fine particles and water swelling, which can reduce the porosity and permeability of the gangue and tailings cemented backfill. Therefore, on the premise of satisfying the flow and mechanical properties of paste backfill, bentonite can be used to improve the permeability of cemented backfill and reduce the leaching and migration of heavy metal ions.

## 1. Introduction

Mining is the basic industry of China’s national economy, which provides important support for the development strategy of national energy and materials [1,2,3,4]. However, more and more attention has been paid to ecological environmental problems such as surface subsidence, groundwater loss, and formation structure destruction during mining [5]. In recent years, backfill mining has been an important development direction of safe and green mining in China, and it has significant advantages in safe mining, solid waste utilization, and ecological environment protection [1,5]. Alternatively, the high cost of backfill materials has become an important reason why it is difficult to promote the application of backfill mining at this stage [6]. Under the severe conditions of the international ecological environment, it is urgent to develop new backfill materials with low cost, and both green and stable performance [7].

In recent years, researchers have combined the reuse of solid waste with backfilling and mining to develop new filling materials such as tailings [8], fly ash [9,10,11,12,13], coal gangue [14,15,16,17,18] and coal gasification slag [6,19,20,21]. The reuse of solid waste can alleviate the cost pressure of backfill mining to a large extent, but most solid waste materials (tailings, coal gangue and coal gasification slag, etc.) as the derivative residues of the chemical and metallurgical industries, contain a large number of heavy metal elements, and cause great problems to the underground environment after backfill [6,9,22]. Therefore, it is urgent to develop a method that can not only meet the flow and mechanical properties of backfill but also reduce the migration or leaching of heavy metal elements when solid waste is reused as backfill materials, to protect the long-term stability of the ecological environment such as groundwater [23].

Permeability reflects the transmission performance of backfill as a porous medium and refers to the difficulty of heavy metal ions in gas or liquid penetrating, diffusing, or migrating to groundwater near the backfill under the action of pressure concentration and other gradients [24]. The penetration performance of the pore structure is the most important index affecting the permeability. The number of connected pores and the size of the pore diameter can provide favorable channels for the transmission of toxic and harmful substances [25,26]. At present, there are few reports on the pore structure and permeability of backfill. Song Xue et al. [27] found that tailings particles generated from metal mines may contain radioactive components and heavy metal ions, and harmful substances will flow out with water seepage around the backfill after they are used in mine backfill, thus causing pollution to the underground environment and even groundwater. Zhang et al. [28] studied the permeability characteristics of backfill and designed and prepared highly durable underground backfill artificial pillars. Qiu et al. [24,29] researched the influence of different waste rock and tailings contents on the strength and permeability of cemented backfill, which revealed that reducing the cement–sand ratio would also reduce its permeability, and found the change rule and characteristics of permeability after cement curing. He et al. [30] investigated the flow properties, mechanical properties and permeability of polypropylene fiber on cemented backfill. Adding fiber to enhance and improve the performance of cemented backfill has certain positive effects on permeability. In contrast, Wu et al. [31] characterized the permeability performance of backfill with different initial temperature, water–cement ratio and curing temperature through numerical simulation, and obtained the permeability evolution rule. The above conclusions are all about the evolution law of the permeability of the backfill under different working conditions. Finding a reasonable pore structure distribution and determining how to reduce the permeability of the cemented backfill are not made.

Bentonite is often discarded as a related impurity in the coal production process and is not further utilized [32,33]. However, bentonite has excellent expansibility and compactness, can fill the micropore structure of concrete, and can also be used as a waterproof material [34]. This is because the volume of bentonite micro-particles becomes larger after water absorption, which closes the transmission channel of permeating gas or liquid, and effectively prevents harmful ions in solid waste backfill materials from migrating or leaching into groundwater [35,36]. Bentonite has great potential to improve the performance of cemented backfill. Therefore, in this study, bentonite is used to improve the flow performance, mechanical properties, pore structure and permeability of gangue cemented backfill and tailing cemented backfill, which is of great significance for the wide application of cemented backfill and the protection of the groundwater underground environment. It lays a foundation for the combination of backfill and mining environmental protection. Therefore, this study proposes a new method to use bentonite to improve the flow properties, mechanical properties, pore structure and permeability of gangue cemented filling bodies and tailing sand cemented filling bodies for preliminary research, and to improve the application performance of new filling materials, which is of great significance for the wide application of cementation filling and underground environmental protection of groundwater, and lays a foundation for the combination of filling and mining environmental protection.

## 2. Materials and Methods

### 2.1. Raw Material Characteristics

**Figure 1:**
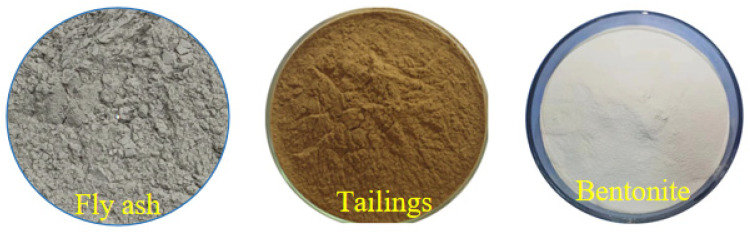
Photos of raw materials.

The filling materials used in this study include ordinary Portland cement, fly ash, coal gangue, and tailings, Figure 1 shows a raw material photo. P.O 42.5 Portland cement was used as cementing material, fly ash (FA) produced by a power plant in Yulin, Shaanxi was used as auxiliary cementing material, and coal gangue and tailing sand produced by a coal mine in Yulin, Shaanxi was used as aggregate. The gangue was screened and crushed, and the particle size was 0~2.5, 2.5~4.0, 4.0~5.0, 5.0~8.0, 8.0~9.5, 9.5~13.2, 13.2~16.0 mm, and the particle size distribution of gangue was controlled by Taylor gradation recombination, and the Taylor series was selected as 0.35. The mass ratio of gangue in seven particle size grades was determined. Remember that the maximum particle size of the particle is *x_max_*, according to the gradation theory, the ratio of the mass *M* of the particle size less than or equal to x in the sample to the total mass of the particle *M_t_*.

$$\frac{M}{M_{t}}={\left(\frac{x}{x_{\max}}\right)}^{n}$$

where *n* is the Taylor index, take 0.35.

If the particle size is located at [*x*_1_,*x*_2_], the particle mass *M*_1_ is

$$M_{1}=\left[{\left(\frac{x_{2}}{x_{\max}}\right)}^{n}\times {\left(\frac{x_{1}}{x_{\max}}\right)}^{n}\right]M_{t}$$

**Figure 2:**
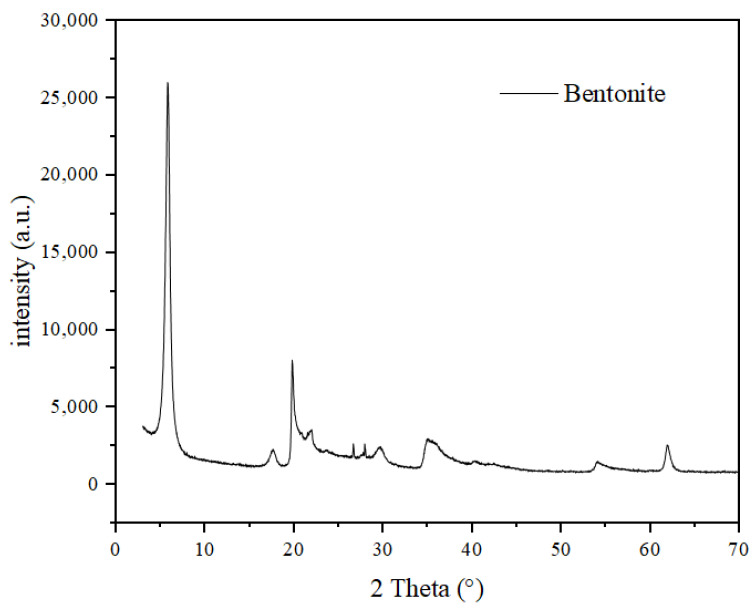
Bentonite X-ray Diffraction.

The tailings were selected from a metal mine in Yulin and the color was yellowish brown. The calcium Bentonite was selected from Nanyang, Henan, China. Its color is grayish white. The particle size distribution has been tested in the range of 10~362 μm, and the density is 2.78 g/m^3^. Bentonite has cohesiveness, expansibility, and impermeability [37]. Due to its special properties, ultra-fine particle size can be used to fill the pore structure of gangue cemented backfill and tailing cemented backfill to improve the mechanical properties and permeability resistance of the backfill. The mixing water was collected from the tap water of Xi’an laboratory in Shaanxi Province. Table 1 shows the chemical composition of P.O 42.5 common Portland cement, fly ash, coal gangue, tailing sand and bentonite. Table 2 shows the mineralogical composition of bentonite. Figure 2 shows the bentonite X-ray diffraction.

### 2.2. Sample Preparation and Maintenance

To meet the requirements of the field pipeline transportation, the mass concentration was set at 75%. Bentonite was selected to replace part of the coal gangue and tailing content in the test, and the test scheme design is shown in Table 3. This experiment adopts the control variable method, keeping the cementitious material and auxiliary cementitious material unchanged, replacing the aggregate with bentonite, and controlling the bentonite content at 0–12%. Before the test began, the dry material was thoroughly stirred and then mixed with stirring water to stir evenly. Part of the slurry was taken for a slump test and bleeding rate test. Finally, the remaining part of the slurry was put into a 50 × 100 standard cylindrical sample, and the mold was removed two days later for standard maintenance (temperature 20 ± 1 °C, humidity 95 ± 1%). A uniaxial compressive strength test and permeability test were carried out when the sample was cured to the design age of the test.

### 2.3. Testing Method

#### 2.3.1. Slump Test

**Figure 3:**
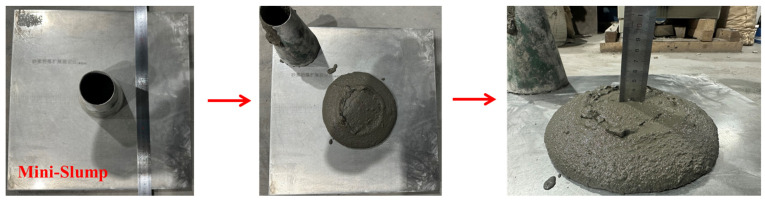
Mini slump test.

Slump is an important index to describe the flow of backfill slurry, and it is also one of the easiest ways to test the flow performance of concrete in engineering practice [38]. The micro-cone slump bucket test was used to characterize the influence of bentonite content on gangue cemented backfill slurry, and the test was carried out according to the GB/T50080 standard of the China National Standardization Committee [39]. The use of a micro-slump bucket can save more test materials, and it can also reliably characterize the flow performance of slurry. Many scholars have used this slump bucket to carry out tests. The dimensions of the miniature slump bucket are as follows: the cone is 150 mm high, the bottom diameter is 100 mm, and the top diameter is 50 mm [38]. Place the steel plate in a horizontal position, wipe the steel plate and the truncated cone circular mold with a damp cloth, and then place the wetted truncated cone circular mold in the center of the steel plate; Quickly pour the prepared filling slurry into the truncated circular mold, and use a spatula to scrape the upper opening flat; After removing the slurry from the glass plate at the edge of the cylinder, lift the truncated circular mold vertically and steadily, while turning on the stopwatch for timing. When the slurry no longer diffuses or the diffusion duration has reached 30 s, use a steel ruler to measure the maximum diameter of the slurry flowing in two perpendicular directions and take the average value (in mm) as the diffusion coefficient of the filling slurry. The test process is shown in Figure 3. Slump value and diffusion coefficient were recorded. The test was repeated three times for each group, and the average value was taken for further analysis.

#### 2.3.2. Bleeding Rate Test

The bleeding rate of the backfill slurry was tested using a 2000 mL measuring cylinder to calculate the percentage of surface water mass in the total mass. Before the test began, the inner wall of the measuring cylinder was moistened with a wet cloth, the stirred slurry was loaded into the measuring cylinder until 2000 mL, and the surface water was removed and weighed after standing for 24 h.

#### 2.3.3. Unconfined Compressive Strength Test

Unconfined compressive strength tests can obtain the strength characteristics of back-fill more conveniently and quickly [40,41]. An MTSC43.504 electronic universal test machine was used to carry out the UCS test according to the GB/T17671-2021 national standard [42]. The equipment is produced by China Changchun Sinotest Equipment Co., Ltd., Changchun, China. The displacement loading method is selected, and the loading speed is set to 1 mm/min. Each group of samples was tested three times, the test data were recorded, and the average strength was taken for further analysis.

#### 2.3.4. Porosity Test

The porosity was tested by nuclear magnetic resonance (NMR) and the MacroMR12-150H-l testing machine was selected. The equipment is manufactured by Suzhou Newman Analytical Instrument Co., Ltd., Suzhou, China. The nuclear magnetic resonance testing technology measures the relaxation characteristics of the fluid in the pores of the filling material based on the interaction between the H proton in the fluid and the external magnetic field. During the experiment, an external magnetic field emits a certain frequency of radio frequency pulses, causing the H protons in the pores of the filling material to magnetize, and finally resonate to absorb energy. After the radio frequency pulse is terminated, the H protons release the absorbed energy, which can be detected by the coil outside the filling material core. The cylindrical sample to be tested was saturated in water for 24 h, the free water on the surface of the sample was wiped dry and wrapped with plastic wrap to prevent water evaporation, and then the nuclear magnetic test was carried out to record the data and calculate the porosity of the backfill sample.

#### 2.3.5. Permeability Test

**Figure 4:**
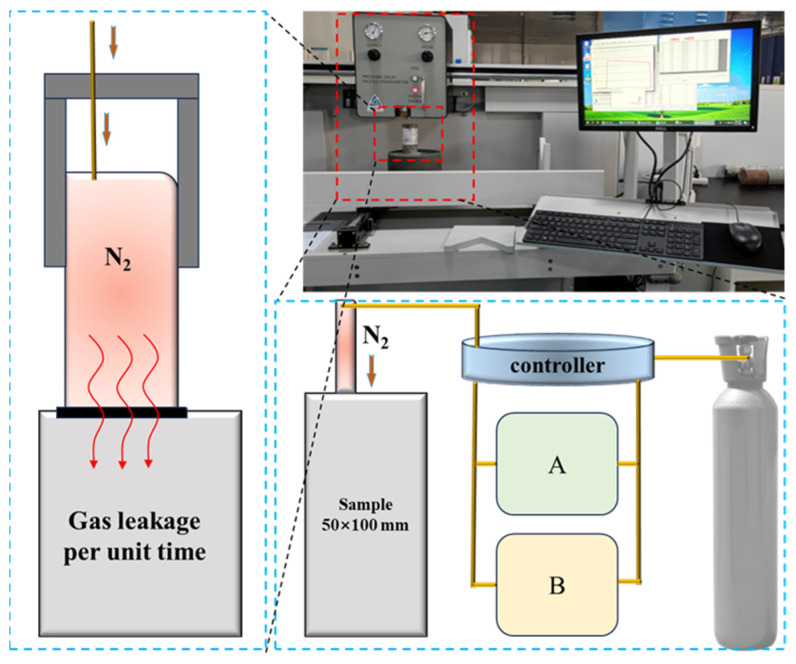
Principle of permeability test.

The permeability of backfill samples was tested by the PDPK-400 pressure attenuation profile permeability meter, and the testing gas medium was nitrogen. The equipment is manufactured by Core Laboratories in Houston, TX, USA. Due to the requirements of the device, each sample was tested six times, the maximum and minimum values were excluded, and the average value of the middle four times was taken as the permeability value of the sample. Figure 4 shows the principle of the permeability test. Please refer to the reference [30] for the specific test principle.

## 3. Results and Discussion

### 3.1. Slump

**Figure 5:**
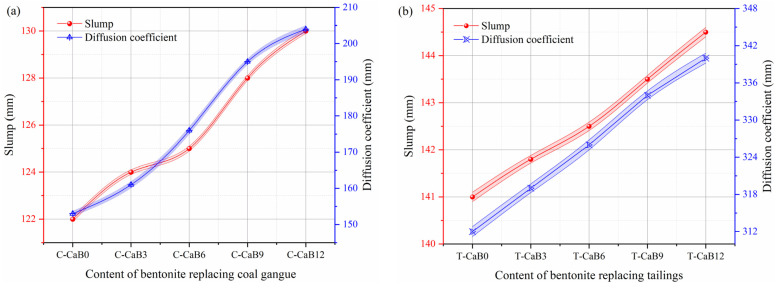
Slump curve of bentonite replacing gangue and tailing with different content. Note: The error line represents the error range obtained by testing multiple sets of test results for the backfill sample. (**a**) Slump curve of bentonite substituted coal gangue content; (**b**) Slump curve of bentonite substituted tailing content.

Under the condition that the mass concentration ratio is 0.75, the slump test results of bentonite replacing gangue and tailing with different contents are shown in Figure 5. Both slump value and diffusion coefficient of slurry of gangue cemented backfill (Figure 5a) and tailings cemented backfill (Figure 5b) gradually increased with the increase of bentonite content. Under different bentonite content, the slump of gangue cemented backfill slurry varied from 122 mm (C-CaB0) to 130 mm (C-CaB12), and the diffusion coefficient varied from 153 mm (C-CaB0) to 204 mm (C-CaB12). The slump of tailings consolidated backfill slurry ranged from 141 mm (T-CaB0) to 144.5 mm (T-CaB12), and the diffusion coefficient ranged from 312 mm (T-CaB0) to 340 mm (T-CaB12). In addition, when no bentonite was used to replace gangue and tailing, the slump values of backfill slurry were 122 mm (C-CaB0) and 141 mm (T-CaB0), and the corresponding diffusion coefficients were 153 mm (C-CaB0) and 312 mm (T-CaB0), respectively. The test results of this study all meet the slump test requirements required for backfill pipeline fluidity, and the minimum slump value is greater than 71 mm [38].

According to the above phenomenon, under the same water–cement ratio, the fluidity of gangue backfill slurry is worse than that of tailing sand backfill slurry, which is due to the large difference in aggregate particle size between gangue and tailing sand. The particle size of the gangue used in the study ranges from 0 to 13.2 mm, and the gangue is an irregular polyhedron with an uneven particle surface, which is easy to produces friction resistance with the gel system of the backfill slurry, which is not conducive to the free flow of the slurry. On the contrary, the particle size distribution of the tailings is uniform, and the particle size distribution range is 0~2.12 mm. In particular, the shape of the tailing sand particles is like a ball, which is easy to roll and slip between the slurry, and the tailing sand is easier to meet the fluidity requirements than the fluidity of the gangue backfill slurry. In addition, the fluidity of the two different aggregate backfill slurryies developed in a good direction when replaced by bentonite, and the fluidity of the gangue backfill slurry was improved more significantly. Bentonite can be filled into the pores of the backfill slurry, reduce the friction resistance generated by the slurry flow, and promote the lubrication of the slurry, thus improving the flow characteristics of the backfill slurry. Bentonite improves slurry fluidity, while the viscosity and slippability of slurry are enhanced by the microbead effect generated by the fine particles of bentonite. Since bentonite particles have a negative charge in the solution, and other ions in the solution are metal cations, the two will attract each other when the surface is in contact, promoting a more uniform distribution of slurry. The above discussion is similar to the results in the literature [43]. In general, bentonite replaces gangue and tailing as backfill material, and its excellent expansibility and compactness can improve the fluidity of backfill slurry to a certain extent, which meets the fluidity requirements of mine backfill slurry.

### 3.2. Bleeding Rate

**Figure 6:**
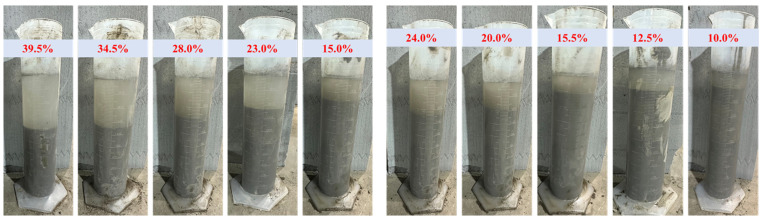
Bleeding rate test result. Note: After standing for 24 h, the ratio of the mass of the slurry after removing the surface water to the total mass of the raw slurry is the bleeding rate, expressed in wt%.

**Figure 7:**
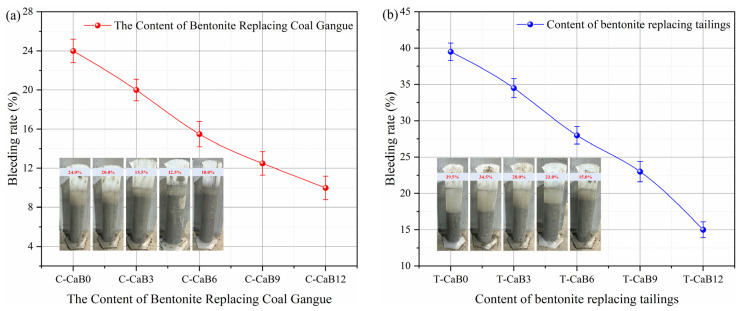
Curve of bleeding rate when bentonite replaces gangue and tailing with different content. Note: The error line represents the error range obtained by testing multiple sets of test results for the backfill sample. (**a**) Bleeding rate curve of bentonite substituted coal gangue content; (**b**) Bleeding rate curve of bentonite substituted tailing content.

Under the condition that the mass concentration is 75%, the test results of the bleeding rate of bentonite replacing gangue and tailing with different contents are shown in Figure 6. The bleeding rate of the slurry of gangue cemented backfill (Figure 7a) and tailings cemented backfill (Figure 7b) decreased gradually with the increase of bentonite content. In the past, gangue and tailing were used in the backfill face, but the strip backfill face would cause slurry leakage due to the high bleeding rate, which seriously restricted the construction progress of the working face. However, the phenomenon that bentonite reduces the bleeding rate of gangue/tailing cemented fill body is beneficial to the strength development of mine backfill materials and site construction. The addition of bentonite can significantly reduce the bleeding rate of backfill slurry. In this experiment, the bleeding rate of gangue/tailing cemented backfill slurry was negatively correlated with bentonite content. The bleeding rate of the slurry of gangue cemented backfill can be reduced from 24.0% to 10.0%. The bleeding rate of tailings consolidated backfill slurry can be reduced from 39.5% to 15.0%.

One of the reasons for the decrease in bleeding rate is that bentonite has certain water absorption and significant water absorption expansion, which can consume the water in the slurry and reduce its water content, thus reducing the bleeding rate of the slurry. Secondly, the saturated bentonite can support and suspend the large particle aggregate, which is precipitated and separated downward in the slurry, so that the particle distribution of the slurry is more uniform, and the strength and permeability of the cured backfill will be improved. In addition, we believe that the microbead effect of bentonite and fly ash fine particles can improve the bonding force between slurry particles [44]. When bentonite is moistened and expanded, its particles will attract one another and form a binding water film when they contact the surface of gangue/tailings particles [45]. The bentonite particles are connected in a chain structure by cations to reduce free water in the slurry, which can significantly reduce the bleeding rate of the filler slurry.

### 3.3. Mechanical Property

**Figure 8:**
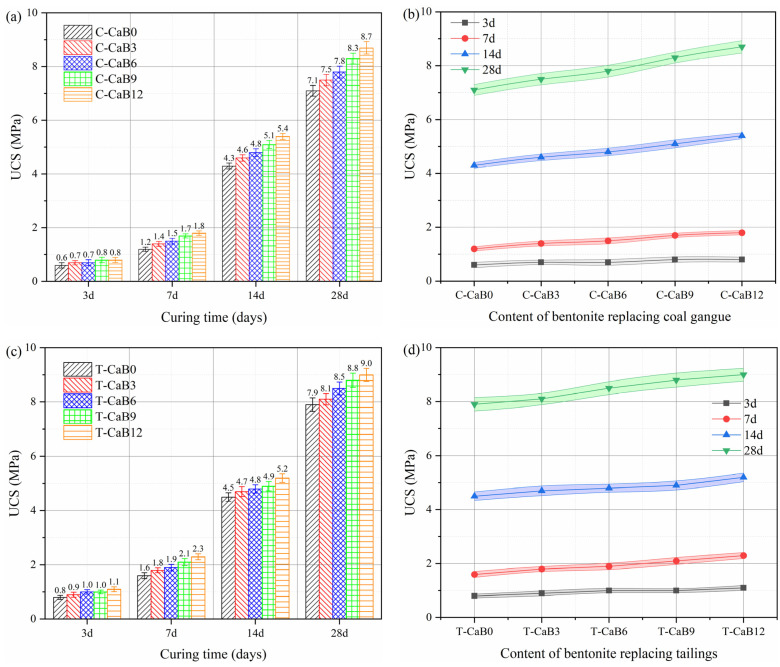
Strength test results of bentonite replacing gangue and tailing sand with different contents. Note: The error line represents the error range obtained by testing multiple sets of test results for the backfill sample. (**a**,**b**) The relationship between strength characteristics and age of bentonite replacing coal gangue with different content; (**c**,**d**) The relationship between strength characteristics and age of bentonite tailings with different content.

Mechanical properties are crucial to the long-term stability of backfill and the effect of backfill treatment. Figure 8 shows the strength test results of bentonite replacing gangue and tailing with different contents. It can be seen from Figure 8a,c that with the increase of curing age, the strength of gangue and tailing cemented backfill increases rapidly with the continuous hydration, and the research results are similar to the previous strength test results of backfill [24]. The development of the above strength with curing age is mainly due to the hydration reaction of cement and the pozzolanic reaction of fly ash. Under the condition that the mass concentration ratio is 0.75, the strength development of tailing cemented backfill is better than that of gangue. The three-day strength of gangue and tailing cemented backfill is 0.6 MPa and 0.8 MPa, respectively, and the 28-day strength is 7.1 MPa and 7.9 MPa, respectively.

When bentonite was added, the strength of gangue and tailing cemented backfill increased significantly, and the strengthening effect became more and more significant with the increase of bentonite content. It can be seen from Figure 8b,d that the strength of cemented backfill increased not significantly at three days after the addition of bentonite, and the strengthening effect was mainly concentrated at 28 days. In conclusion, the addition of bentonite can enhance the strength properties of both gangue and tailing cemented backfill. This is mainly due to the swelling property of bentonite in contact with water, which can be filled into the pore structure of the cemented backfill [46]. Fly ash and bentonite both have an enhancement effect on strength development, but the enhancement mechanism is different. In the early hydration process, Ca^2+^, Mg^2+^, and a large amount of OH^−^ are produced, which makes the pH of the whole system rise rapidly, accelerates the dissolution of silicate and aluminate on the surface of fly ash particles, and forms a plasma liquid phase reaction system. Hydration products such as Ca(OH)_2_ and C-S-H gel are generated through the interaction between ions, which contributes to the hydration reaction of cement, and also stimulates the pozzolanic reactivity of fly ash [47,48].

In addition, bentonite has a higher content of spherical particles, and when mixed with cement, it has a heterogeneous nucleation effect, which can provide more nucleation sites for the hydration products in the gangue/tailing filler slurry, thus promoting the hydration reaction. In the early stage, due to the rapid hydration reaction, it played a major role in the strength development of the backfill, but compared with the pore back-fill effect of bentonite, the enhancement effect was not significant. In the later reaction of volcanic ash, the hydration reaction is weakened, and the effect of alkali excitation of fly ash and backfill of bentonite is more significant [49,50,51]. Among them, bentonite is filled with spherical particles in the backfill pore structure, thus enhancing its strength development. With the increase in bentonite content, its backfill effect is more obvious and its strength is better [52]. Therefore, bentonite can promote and improve the development of backfill strength and is a good mineral additive in the mining backfill industry.

### 3.4. Porosity and Permeability

At present, the gangue produced by coal mining is widely used in the field of mine backfill, but the potential pollution risk of heavy metal ions inside the gangue to the water resources in the mining area is becoming more and more prominent. As a major source of waste from metal mining, tailings contain a much higher heavy metal content than coal gangue—which is widely used in mine backfill—and will have higher potential risks. Gangue and tailing cemented backfill are porous media materials, heavy metal ions will migrate down through the pore structure of the backfill, and the underground ecological environment will face severe challenges [53,54]. Therefore, how to permanently fix the heavy metal elements in the solid waste of gangue and tailings inside the backfill body has become a practical and effective path to solve the sustainable development of solid waste backfill materials. On the premise of satisfying the above flow and mechanical properties, the mineral additive bentonite was used in this study to reduce the porosity and permeability of the backfill.

**Figure 9:**
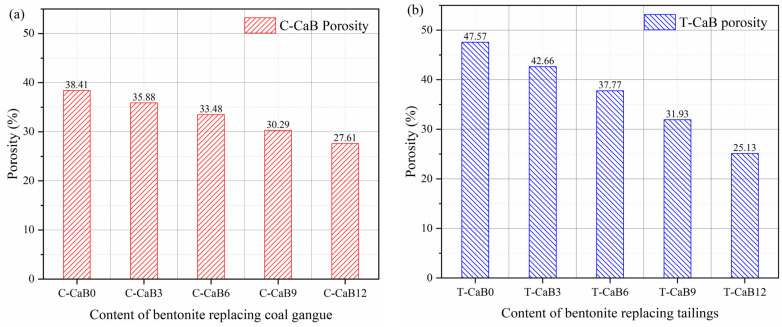
Porosity test results of bentonite replacing gangue and tailing with different contents. (**a**) Porosity test results of bentonite replacing coal gangue with different content; (**b**) Porosity test results of bentonite substituting tailings with different content.

Figure 9 describes the porosity test results of bentonite replacing gangue and tailing with different contents. When the curing age reaches 28 days, the porosity of gangue and tailing cemented backfill without bentonite is 38.41% (C-CaB0) and 47.57% (T-CaB0), respectively, and the porosity of tailing cemented backfill is much higher than that of gangue cemented backfill. The particle size mainly derived from tailings is much smaller than that of gangue. In fact, under the same volume demand, due to the relatively high volume proportion of large gangue particles, the pore structure between particles is less, and the overall porosity will be lower. Relatively speaking, the demand for tailings articles will be higher, which will increase the porosity of the backfill. With the increase of ben-tonite content, the porosity of the two cemented backfills gradually decreased to 27.61% (C-CaB12) and 25.13% (T-CaB12), respectively. The backfill effect of the mineral additive bentonite can be well demonstrated by the change of porosity. This fills the pore structure of the backfill and assists the hydration products to glue tailings/gangue aggregate particles, thereby reducing the primary porosity. In addition, compaction occurs when the backfill is subjected to top pressure in the formation structure [55]. The main reason for this process is that the backfill will compress and deform when subjected to low-level pressure, which promotes the mutual extrusion of particles and makes the microstructure of the backfill more dense [56].

In theory, the compaction process can reduce the primary porosity of the backfill to a certain extent, which is beneficial to the long-term development of the backfill [57]. However, some studies have shown that although the compaction process will reduce the porosity of the backfill, it will also make the internal pores of the primary backfill penetrate each other, thus increasing the permeability of the backfill. Therefore, porosity alone cannot be used to describe heavy metal migration in backfill.

**Figure 10:**
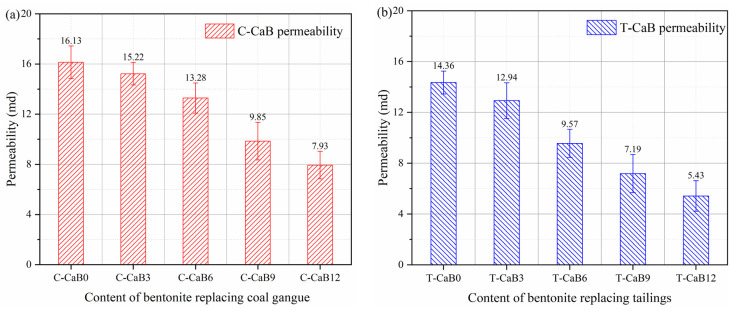
Permeability test results of bentonite replacing gangue and tailing with different contents. (**a**) Permeability test results of bentonite replacing coal gangue with different content; (**b**) Permeability test results of bentonite substituting tailings with different contents.

Permeability can be used to describe the ability of backfill to allow fluid to pass through and is one of the important indicators to demonstrate the improvement of ben-tonite performance of gangue and tailing cemented backfill. Figure 10 describes the permeability test results of bentonite replacing gangue and tailing with different contents. The permeability of gangue and tailing cemented backfill samples without bentonite as a mineral additive is 16.13 md (C-CaB0) and 14.36 md (T-CaB0), respectively. It can be observed that the permeability of gangue cemented backfill varies from 16.13 md (C-CaB0) to 7.93 md (C-CaB0) with the increase of bentonite content, and the permeability of tailing cemented backfill varies from 14.36 md (C-CaB0) to 5.43 md (C-CaB0). The addition of bentonite can significantly improve the permeability of cemented backfill. Bentonite compacts the backfill structure by backfilling physical pores, thus reducing the permeability of the backfill. For gangue cemented backfill materials, gangue particles and bentonite cemented materials occupy a large area on the cross-section of the backfill through the micro-expansion of bentonite wetting, and the cross-sectional area of gas passing through the backfill sample quickly is small in the permeability test. As for the tailings cemented backfill material, although the particle size is small, the adhesive force with the cementing material is better [58]. The addition of bentonite can more fully wrap the aggregate particles and reduce the connectivity of pores. Therefore, it is recommended to add bentonite in engineering applications to improve the performance of backfill.

## 4. Conclusions

In order to protect the sustainable development of underground ecological environment and meet the paste backfill index, bentonite is used to improve the permeability of cemented backfill and reduce the leaching and migration of heavy metal ions. In this study, in order to better explore the improvement effect of bentonite on the performance of gangue/tailing cemented backfill, the fluidity test, mechanical property test and permeability test were carried out. Through experimental analysis, slump, diffusion diameter and bleeding rate are used to describe the flow properties of the cemented backfill, uniaxial compressive strength is used to describe the mechanical properties of the cemented backfill, and porosity and permeability are used to describe the permeability of the cemented backfill. Based on the above test results, the following conclusions can be drawn:

(1) After the addition of bentonite into the gangue/tailings consolidated backfill, the bleeding rate is significantly reduced, while the slump value is increased and the fluidity is improved. The bleeding rate of gangue and tailing cemented backfill decreased to 10% and 15%, respectively, and slump value increased to 130 mm and 144.5 mm, respectively. Bentonite can improve the fluidity of cemented backfill, especially its bleeding rate, and promote the long-term development of cemented backfill;
(2) After adding bentonite into the gangue/tailing cemented backfill, the mechanical properties are significantly improved. The early improvement effect was not obvious, mainly concentrated in the middle and late curing of cemented backfill. With the increase of bentonite content, the 28-day uniaxial compressive strength increased from 7.1 MPa and 7.9 MPa to 8.7 MPa and 9.0 MPa, respectively.
(3) Bentonite is filled between the pores of the cemented backfill with its fine particles and swelling in water, which can reduce the porosity of both gangue and tailings cemented backfill. The porosity of gangue cemented backfill decreased from 38.41% to 27.61%, and the porosity of tailing cemented backfill decreased from 47.57% to 25.13%;
(4) Bentonite has a significant improvement effect on the permeability of gangue and tailing cemented backfill. Bentonite and cementified material can produce better adhesive force, fill the pore structure, reduce the porosity through, and thus reduce the permeability of cemented backfill. In engineering application, it will effectively prevent the leaching and migration of heavy metal ions in the filled aggregate.

## Figures and Tables

**Table 1:** Chemical constituents of materials, wt%.

Composition	CaO	SiO_2_	MgO	Fe_2_O_3_	Al_2_O_3_	SO_3_	TiO_2_	Loss
Cement	50.8	26.45	4.58	3.01	7.94	1.18	0.31	5.73
FA	15.13	40.12	1.29	13.15	16.24	4.91	1.01	8.15
Coal Gangue	1.79	59.94	1.78	7.59	22.15	0.61	0.15	5.99
Tailings	0.67	71.11	0.11	0.51	14.03	0.23	0.01	13.33
Bentonite	3.41	63.52	2.83	6.07	15.44	0.03	0.99	7.71

**Table 2:** Mineralogical composition of bentonite, wt%.

Sample Name	Montmorillonite	Quartz	Quartz	Albite
Bentonite	96.20	0.60	2.10	1.10

**Table 3:** Experimental scheme, g.

No.	Alternative	Aggregate	FA	Cement	Bentonite	Water
C-CaB0	Coal Gangue	620	200	180	0	334
C-CaB3		590			30	
C-CaB6		560			60	
C-CaB9		530			90	
C-CaB12		500			120	
T-CaB0	Tailings	620	200	180	0	334
T-CaB3		590			30	
T-CaB6		560			60	
T-CaB9		530			90	
T-CaB12		500			120	

Note: CaB represents the content of bentonite, C represents coal gangue, and T represents tailings.

## Data Availability

The data used to support the findings of this study are included in the article.
